# Snat: a SNP annotation tool for bovine by integrating various sources of genomic information

**DOI:** 10.1186/1471-2156-12-85

**Published:** 2011-10-07

**Authors:** Jicai Jiang, Li Jiang, Bin Zhou, Weixuan Fu, Jian-Feng Liu, Qin Zhang

**Affiliations:** 1 Key Laboratory of Animal Genetics and Breeding of the Ministry of Agriculture, College of Animal Science and Technology, China Agricultural University, Beijing, 100193, P.R. China

## Abstract

**Background:**

Most recently, with maturing of bovine genome sequencing and high throughput SNP genotyping technologies, a large number of significant SNPs associated with economic important traits can be identified by genome-wide association studies (GWAS). To further determine true association findings in GWAS, the common strategy is to sift out most promising SNPs for follow-up replication studies. Hence it is crucial to explore the functional significance of the candidate SNPs in order to screen and select the potential functional ones. To systematically prioritize these statistically significant SNPs and facilitate follow-up replication studies, we developed a bovine SNP annotation tool (Snat) based on a web interface.

**Results:**

With Snat, various sources of genomic information are integrated and retrieved from several leading online databases, including SNP information from dbSNP, gene information from Entrez Gene, protein features from UniProt, linkage information from AnimalQTLdb, conserved elements from UCSC Genome Browser Database and gene functions from Gene Ontology (GO), KEGG PATHWAY and Online Mendelian Inheritance in Animals (OMIA). Snat provides two different applications, including a CGI-based web utility and a command-line version, to access the integrated database, target any single nucleotide loci of interest and perform multi-level functional annotations. For further validation of the practical significance of our study, SNPs involved in two commercial bovine SNP chips, *i.e*., the Affymetrix Bovine 10K chip array and the Illumina 50K chip array, have been annotated by Snat, and the corresponding outputs can be directly downloaded from Snat website. Furthermore, a real dataset involving 20 identified SNPs associated with milk yield in our recent GWAS was employed to demonstrate the practical significance of Snat.

**Conclusions:**

To our best knowledge, Snat is one of first tools focusing on SNP annotation for livestock. Snat confers researchers with a convenient and powerful platform to aid functional analyses and accurate evaluation on genes/variants related to SNPs, and facilitates follow-up replication studies in the post-GWAS era.

## Background

Currently, genome wide association studies (GWAS) have been widely accepted as a primary approach for gene identification concerning complex traits. A subset of SNPs related to the trait of interest can be derived from GWAS at a specified level of statistical significance. To further determine true association findings in GWAS, the common strategy is to sift out the most promising SNPs for follow-up replication studies. Hence it is crucial to explore the functional significance of the candidate SNPs in order to screen and select the potential functional ones. So far, a variety of public bioinformatics databases, *e.g*., NCBI Entrez Gene [1], UniProt [2], Gene Ontology [3], KEGG PATHWAY [4] and AnimalQTLdb [5], *etc*., contain different aspects of biological information required for SNP functional annotation. However, it is infeasible to mine the relevant research data from these public sources by a single query. Furthermore, it is a daunting task to integrate various sources of functional information of interest among the large assortment of data in a manual fashion.

To deal with SNP annotation, a number of bioinformatics tools have been created. However, the majority of these tools are for humans, *e.g*., SNPit [6], SNPnexus [7], and few tools are available for other species such as bovine. FunctSNP [8] is currently the only tool available for bovine SNP annotation. Although FunctSNP provides various functions to search and manage annotated data related to SNPs, the main limitations maybe exist: Firstly, it is not straightforward and convenient to annotate even one SNP using FunctSNP, because users should initially construct a local database before SNP annotation and cannot be run online through a web interface, leading to low efficiency and time consuming. Secondly, FunctSNP merely outputs some identifiers relating to some public databases in queries, *e.g*. GO:0000122 (GO term), bta00340 (KEGG Pathway entry), A7YWP4 (UniProt accession), and more detailed descriptions for these identifiers should be obtained manually via accessing the relevant public domains. Finally, FunctSNP only recognizes the cluster ID (rs#) and accurate position of a SNP in dbSNP, triggering an obviously application limitation since users are usually interested in those SNPs without specific cluster IDs or not included in dbSNP in most cases, such that FunctSNP can not deal with these SNPs under this situation.

Focusing on tackling the above limitations, we developed a SNP annotation tool (Snat) to provide a wide array of functional SNP annotations for bovine. In Snat, the most novelty is that recent versions of information from FTPs and webpages of the public domains have been extracted and integrated to construct a composite database. Furthermore, SNPs at arbitrary positions can be well annotated through a single query, regardless of whether they are involved in the dbSNP database or not.

## Implementation

Snat aims at the design for a novel query scheme which can provide precise and comprehensive annotation for bovine SNPs. This would be helpful for mining potential clues of functional importance. Snat is written in Perl as well as SQL scripts with modular architecture. The design of the query scheme with common interfaces supports multiple options for each annotation task.

**Figure 1:**
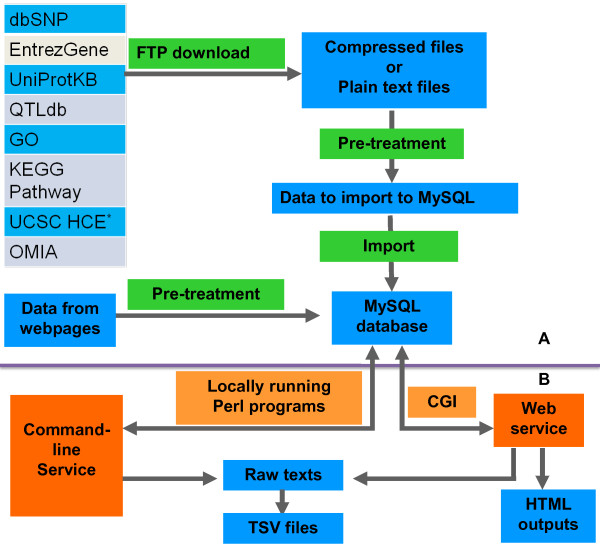
**Construction and implementation of Snat**. This figure shows the construction and implementation of Snat. A) Data from several public biological databases are retrieved by FTP download as well as webpage accessing, then pre-treated to import to MySQL database. B) Web services and locally running programs are constructed. *HCE is short for short for highly conserved element.

**Figure 2:**
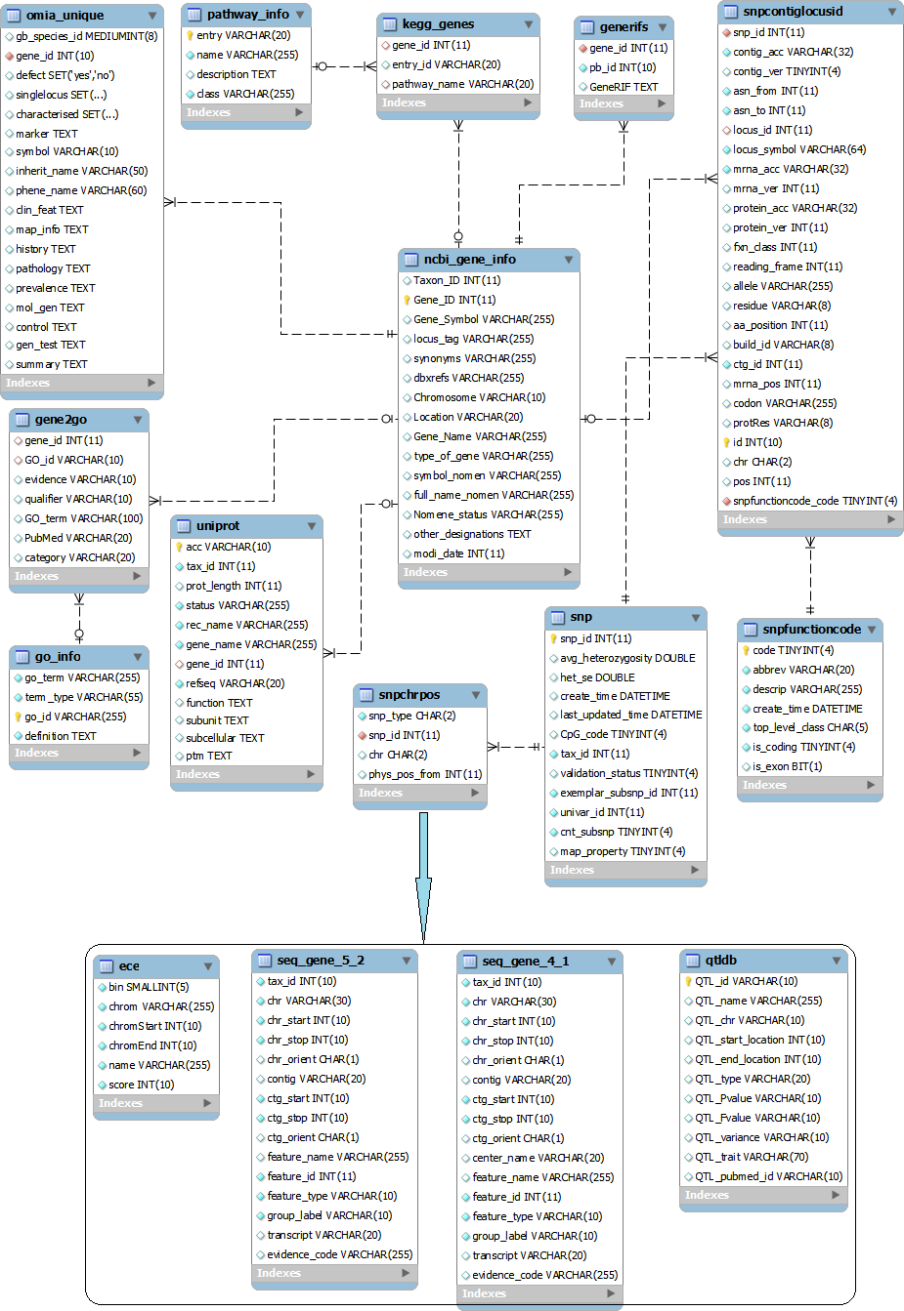
**The schema of the database of Snat**. The schema shows that all the tables and relationships among them in Snat database.

Developing of Snat consists of two major steps: The first step is the construction of the local database that contains substantial information for SNP annotation. Specifically, documents are retrieved from various aspects of online databases using an automated procedure via Perl program. These online resources include dbSNP [9], Entrez Gene, UniProt, GO, KEGG PATHWAY, AnimalQTLdb, UCSC ConsElements [10,11] and OMIA [12]. In order to ensure the accuracy of the online information, Snat integrates packaged data downloaded from FTPs as well as data from webpages (see Figure 1 and Figure 2 for detailed architecture). Subsequently, a local MySQL relational database can be constructed from the retrieved information via SQL and Perl scripts. The second step is the design of a user friendly client-side which can implement SNP annotation with multiple choices. Programs are written in Perl. Users can browse website of Snat to perform online SNP annotation. The annotation results can be viewed in colored and aligned HTML tables, as well as printed in a plain text and downloaded as a text or compressed file.

Besides web-based application of Snat, we also developed an alternative version of Snat in command-line mode, which can be run locally to query and annotate SNPs. Specifically, via downloading the integrated database from the Snat site as a SQL script, users can construct the local database, which is identical with that of web-based version. Based on the local database, SNP annotation can be conducted to print results into a text file via Perl scripts in a command-line fashion. The raw text report, generated by either the locally running Perl programs or downloaded from Snat website, looks not well-formatted but is created with a specified access rule. Thus it can be readily processed via running a Perl program provided by Snat website to produce annotation data structured in TSV files for further analyses.

## Results

### Features

**Figure 3:**
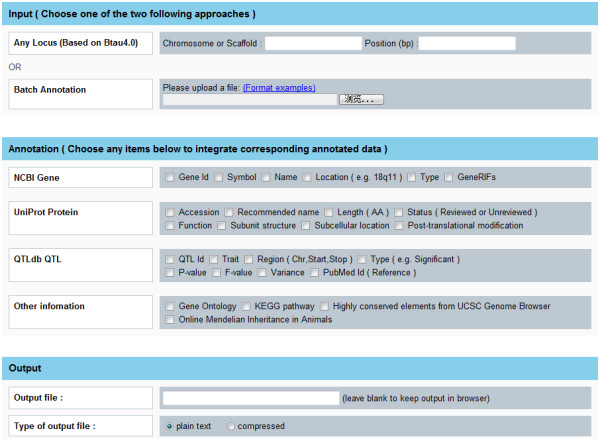
**Web interface of Snat**. The web interface of Snat contains three parts: input, annotation and output.

Snat addresses two functions. One is to query and annotate those SNPs involved in dbSNP, the other is to deal with arbitrary single nucleotide loci no matter whether they are included in dbSNP or not (see Additional file 1). The interface of Snat for web services consists of three parts: input, annotation options and output options (Figure 3). Users can input either coordinate or rs-identifier of individual SNP, or upload an index file containing a bunch of SNPs in a batch fashion, to perform annotation. By setting specified query options, Snat generates corresponding annotated data with combination of various assortments of biological information of SNPs. The outputs of annotation can be browsed online in HTML or integrated in a plain text file for downloading. Alternatively, Snat provides users with Perl programs running in command-line mode to finish annotation locally. Several options can be set for command-line application. The raw text generated by the locally running annotation programs is identical with that downloaded from online annotation. Compared with outputs in HTML, those stored in the raw text are not well-structured. However, the raw text report is created with a specified format which is readily further processed with programming. A corresponding Perl program has been developed to transform the original text to well-formatted data in TSV files that can be clearly viewed by Microsoft Excel and other spreadsheet programs.

Due to incorporating various sources of existing public data into a retrieved database, it is feasible and efficient to obtain annotation data automatically via merely one query in Snat, with seldom need of further re-querying the original public databases manually, facilitating users to rapidly pinpoint these SNPs with functional significance. For further demonstrating the features of Snat, systematic comparisons between Snat and a published tool FunctSNP have been conducted in the following aspects.

First, FunctSNP can only recognize the cluster ID (rs#) or accurate coordinate of a SNP in dbSNP while Snat can deal with an ambiguous coordinate via searching for the nearest SNP or SNPs within a specified distance away from it (see the first example in Additional file 2).

Second, FunctSNP can only run locally and users should construct a local database before SNP annotation, while Snat provides a locally running command-line version as well as online annotation services. Furthermore, FunctSNP outputs annotation information step by step while Snat produces all output data in a single query (see the second example in Additional file 2).

Third, compared with FunctSNP, Snat integrates extra information of GeneRIFs and conserved elements. Additionally, Snat integrates more detailed information for terms of UniProt Protein, GO, KEGG Pathway and QTL than FunctSNP. Specifically, FunctSNP merely outputs the protein accession and name from UniProt while Snat can output its accession, name, length, function, post-translational modification, *etc*.

### Command-line mode

Online annotation works well for submitting dozens or hundreds of SNPs in a single query. However, when users need to annotate thousands of SNPs, it is more efficient to use the locally running command-line mode of Snat. Via downloading the integrated database from the Snat site as a SQL script, users can construct the local annotation database. Two Perl programs, named dbSNP.pl and anylocus.pl given by Snat website, can achieve the function of local running. The programs dbSNP.pl and anylocus.pl play the same roles with the modules of "Search SNPs" and "Any Locus" on the webpage, respectively. For demonstrating the application features of local running of Snat, three examples based on command-line mode for performing annotation are given below:

$ dbSNP.pl --db_name *db_name *--db_user *db_user *--db_password *db_pw *--rs_id *rs109234250 *--all *ex1.raw*

$ anylocus.pl -db_name *name *-db_user *user *-db_password *pw *-chr_pos *14 440000 *-gene *symbol name *-uniprot *acc function *-go *outputfile*

$ dbSNP.pl -db_name *name *-db_user *user *-db_password *pw *-input *inputfile *-gene *gene_id symbol name generifs *-go -kegg -omia -option *30k outputfile*

The command line options db_name and db_user, db_password denote the name, the user name and the password of the local annotation database respectively. Input data can be rs-identifier (for example, --rs_id *rs109234250 *in the first command), SNP coordinate (for example, -chr_pos *14 440000 *in the second command) or an index file that contains numbers of SNP coordinates (for example, -input *inputfile *in the third command). Output reports are generated corresponding to the annotation options in the command. For examples, the option --all means that all annotation information should be included in the output report (see the first command); "-gene *symbol name *-uniprot *acc function *-go" means that the symbol and name of gene, the accession and function of UniProt protein and GO information should be included in the output report (see the second command).

### Output

**Figure 4:**
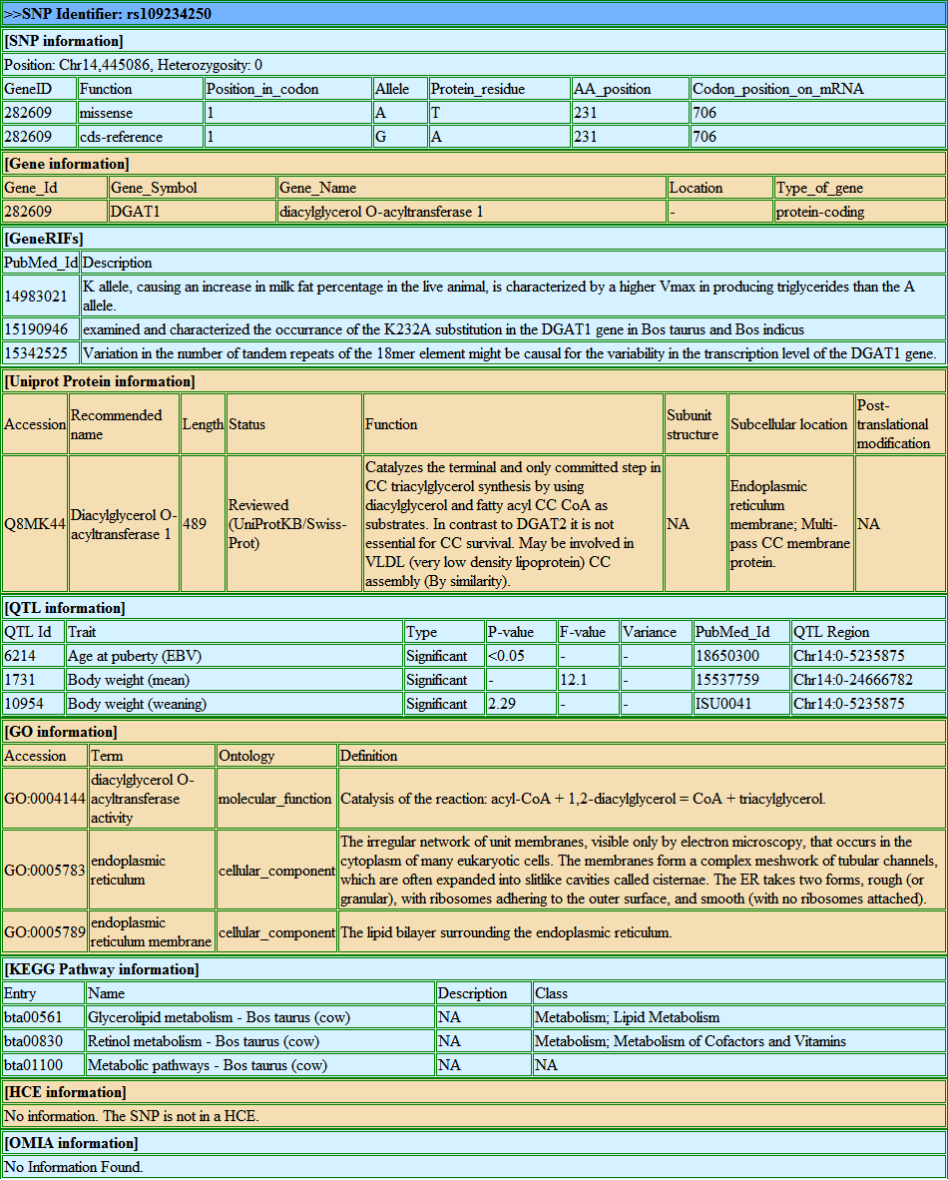
**An example of HTML output**. The first line is coloured blue, showing the items of the query. The following subsections are coloured light green or light red alternately, including SNP information, Gene information, GeneRIFs, Uniprot Protein information, QTL information, GO information, KEGG Pathway information, HCE information and OMIA information.

Snat provides output reports in two different formats, *i.e*., the HTML webpage and the plain text. The HTML output can be clearly viewed online in colored and aligned tables (see Figure 4) while the plain text report is created with a specified format (see Additional file 3). For enhancing readability of annotation data in raw plain text, a Perl program named raw2TSV.pl has been developed to transform the original plain text into several subdata structured in TSV format. An example on how to further process the raw plain text has been given in the following Application subsection.

### Application

For illustrating practical significance of Snat in facilitating functional analyses on genes/variants in the post-GWAS era for bovine, Snat was implemented to annotate 20 identified significant SNPs (Additional file 4) associated with milk yield in our recent GWAS [13]. Various genomic information data (*e.g*., NCBI Gene, UniProt, QTLdb, *etc*.) related to the SNPs of interest as well as all other SNPs within 5k bp region away from these 20 SNPs were mined and integrated into a raw text (Additional file 5). The raw annotation results were then processed by the Perl program raw2TSV.pl to generate a summary report (see Additional files 6,7) and five subdata files (see Additional files 8,9,10,11,12). From Additional file 7, it can be found that within the 5K bp region from the loci Chr14,76703 and Chr14,443937, a number of novel SNPs (see Additional file 8) are present. Among these SNPs several are either missense mutations or harbored in UTR. Moreover, kinds of functional information about genes around the significant SNPs from UniProt, GO and KEGG Pathway further demonstrate that these statistically significant SNPs also have potentially functional significance. For example, with respect to the locus "Chr14,443937", the annotation data of corresponding protein Q8MK44 show "May be involved in VLDL (very low density lipoprotein) CC assembly" (see Additional file 9), and the information of the corresponding pathway bta00561 shows "Glycerolipid metabolism" (see Additional file 11). All the functional information suggests that the identified SNP "Chr14,443937" likely affects milk production traits and merits follow-up functional validation study. In addition, four QTLs (Additional file 12) related to the significant SNP are associated with milk yield, further showing consistent findings with previous QTL mapping studies.

So far, several dense SNP panels, including the Affymetrix Bovine 10K chip array and the Illumina 50K chip array, have been increasingly implemented to identify causal mutations for economic importance in bovine under the framework of GWAS [13,14]. However, biological information related to these SNPs has not yet been provided by the chip manufacturers. It is quite daunting for researchers to conduct SNP annotation via querying various public resources manually SNP by SNP. To aid functional analyses and accurate evaluation on genes related to SNPs in GWAS, Snat was implemented herein to create SNP annotation files containing all SNPs corresponding to two commercial SNP chips. The biological information on each of these SNPs, including SNP information from dbSNP, gene information from Entrez Gene, protein features from UniProt, gene function annotations from GO, KEGG PATHWAY and OMIA, linkage information from AnimalQTLdb and conserved elements from UCSC Genome Browser Database, are integrated in these output files, which are publicly available for downloading from Snat site.

### Running speed

To explore the practical feasibility of Snat, several SNP queries under different scenarios are performed for testing running speed of online annotation.

**Figure 5:**
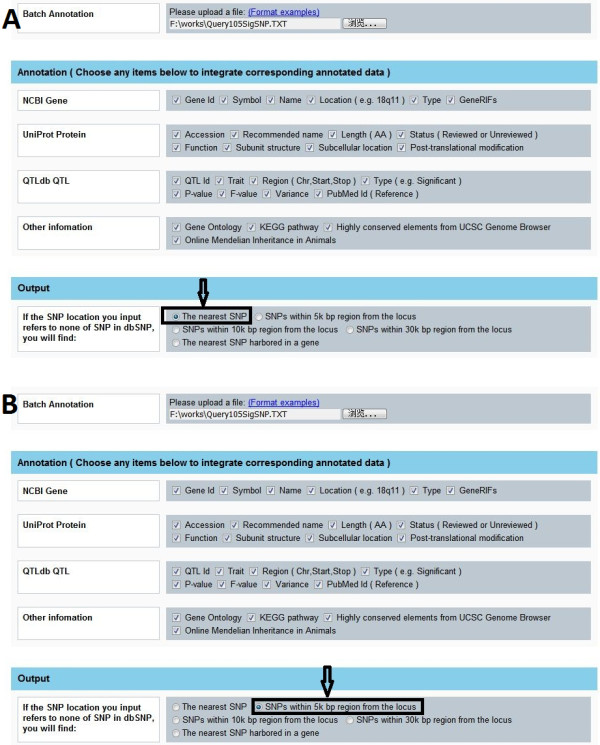
**Screen shots of selected options**. (A) Annotation options are all selected and the option "The nearest SNP" is specified. (B) Annotation options are all selected and the option "SNPs within 5k bp region from the locus" is specified.

For a single SNP annotation, Snat generates outputs immediately once submitting the task. For batch annotation, 105 SNPs (Additional file 13) associated with milk production traits in our earlier GWAS [13] are adopted as the input dataset. With all query options selected as well as the option "The nearest SNP" specified (Figure 5A), it takes about 40 seconds to finish the annotation. When the output option "SNPs within 5k bp region from the locus" specified (Figure 5B), it takes about five minutes to achieve batch annotation of 105 SNPs. It is notable that longer time consumed for the option "SNPs within 5k bp region from the locus" is due to much more SNPs annotated in such situation.

Based on the above tests, the speed of online annotation is practically acceptable as running Snat.

## Conclusions

We developed a SNP annotation tool which can provide comprehensive biological information related to arbitrary single nucleotide position across bovine genome. To our best knowledge, this is one of first tools focusing on SNP annotation for livestock except those for humans. Due to incorporating various sources of existing public data into a retrieved database, Snat provides an efficient and concise way for researchers to obtain annotated data of SNPs automatically via merely one query, with seldom need of further re-querying the original public databases manually.

As known to us, traditional GWAS focus on identification of statistical significance of SNPs with limited understanding of functional aspects of SNPs concerning complex traits. It is a pressing need to make an essential bridge between the outcomes from GWAS and the potential information on the function of genes and pathways. Snat offers an opportunity to achieve this goal. By Snat, researchers can sift out those SNPs indentified in GWAS with functional significance of the complex trait of interest in bovine.

We conclude that Snat can act as a complementary tool for aiding further functional analyses on genes/variants and facilitating follow-up replication studies in the post-GWAS era for bovine.

## Availability and requirements

**Project name**: Snat

**Project home page**: http://animalgenetics.cau.edu.cn/snat/

**Operating system**(s): Any operating system supporting Perl and MySQL

**Programming language**: Perl, SQL

**Other requirements**: Perl DBI and DBD-mysql module

**License**: Free for non-commercial usage

## List of abbreviations

SNP: Single Nucleotide Polymorphism; CGI: Common Gate Interface; HTML: Hypertext Markup Language; TSV: Tab Separated Values; UTR: Untranslated Region

## Authors' contributions

JJ and JL constructed the software and drafted the manuscript. LJ, BZ and WF participated in database construction. JL and QZ designed and supervised the project. All authors read and approved the final manuscript.

## Supplementary Material

Additional file 1 **411 SNPs involved in Illumina Bovine 50K SNP chip but not included in dbSNP**. In Illumina Bovine 50K SNP chip, 52,255 markers are explicitly located in chromosomes based on Btau4.0. However, 411 out of these 52255 SNP markers are not included in dbSNP via position comparison based on Btau4.0. All these 411 SNPs are listed in the table. These SNPs can be annotated through "Any locus" function by Snat. Click here for file

Additional file 2 **Two examples given to compare Snat with FunctSNP**. The examples show the differences between the two tools on features of input data and annotation process. Click here for file

Additional file 3 **Detailed descriptions on annotation results by Snat**. An example is provided to give a detailed explanation on the annotation reports. Click here for file

Additional file 4 **Information of 20 significant SNPs identified associated with milk yield**. This table lists positions of 20 significant SNPs based on Btau4.0 identified associated with milk yield from our recent GWAS results (Jiang et al., 2010). Click here for file

Additional file 5 **The raw annotation outputs for the 20 significant SNPs using Snat**. SNPs within 5k bp region away from these 20 SNPs are annotated by Snat. All the data are printed into the raw text file. Click here for file

Additional file 6 **A summary of annotation data for the 20 significant SNPs structured in TSV format**. The raw annotation outputs of the 20 significant SNPs are further processed to generate a well-structured summary file in TSV format by the program raw2TSV.pl. Click here for file

Additional file 7 **Annotation results of the 20 significant SNPs saved in a worksheet**. The summaries of annotation data for the 20 SNPs structured in TSV format are saved in the worksheet for more easily viewing. Click here for file

Additional file 8 **SNPs involved in dbSNP related to each of the 20 SNPs annotated**. The cluster IDs (rs#) of all SNPs harbored within 5k bp regions from each of these 20 SNPs are integrated. This file is extracted by the program raw2TSV.pl from the raw text file. Click here for file

Additional file 9 **Non-redundant UniProt protein information extracted from the raw annotation outputs for the 20 SNPs**. The file is generated by the program raw2TSV.pl and contains non-redundant UniProt protein information extracted from the raw annotation outputs for the 20 SNPs. Click here for file

Additional file 10 **Non-redundant GO terms extracted from the raw annotation outputs for the 20 SNPs**. The file is generated by the program raw2TSV.pl and contains non-redundant data of GO terms extracted from the raw annotation outputs for the 20 SNPs. Click here for file

Additional file 11 **Non-redundant KEGG Pathway information extracted from the raw annotation outputs for the 20 SNPs**. The file is generated by the program raw2TSV.pl and contains non-redundant KEGG Pathway information extracted from the raw annotation outputs for the 20 SNPs. Click here for file

Additional file 12 **The numbers of QTLs and five most relevant traits corresponding to each of the 20 SNPs**. The file is generated by the program raw2TSV.pl and contains the data of the numbers of QTLs and five most relevant traits corresponding to each of the 20 SNPs extracted from the raw annotation outputs. Click here for file

Additional file 13 **Information of 105 significant SNPs identified associated with milk production traits based on Btau4.0**. This table lists positions of 105 significant SNPs based on Btau4.0 identified associated with milk production traits from our recent GWAS results (Jiang et al., 2010). Click here for file
