# Supplementary material for: Snat: a SNP annotation tool for bovine by integrating various sources of genomic information
Source: BMC Genet. 2011 Oct 7;12:85. doi: 10.1186/1471-2156-12-85 (PMC3224132; doi:10.1186/1471-2156-12-85)
Supplement: Additional file 2 — Two examples given to compare Snat with FunctSNP. The examples show the differences between the two tools on features of input data and annotation process. [file 1471-2156-12-85-S2.PDF]

A. Two examples given to compare Snat with FunctSNP

A1. Example 1: about processing of input data

We annotated the locus “Chr29, 265773” using FunctSNP and Snat respectively. The running commands and results are listed in the following text box under the environment of FunctSNP.

```
1	> loc_1 <- c(265773)
2	> snp_loc_1 <- getSNPID (loc_1, id.type="loc")
3	Species used =    Bos taurus
4	> snp_loc_1
5	Gene_ID     SNP_ID Location
6	1    519580 41576734     265773
7	> geneid_1 <- getGeneID (loc_1, id.type="loc")
8	Species used =    Bos taurus
9	> geneid_1
10	Gene_ID     SNP_ID Location
11	1    519580 41576734     265773
12	>
13	> loc_2 <- c(265772)
14	> snp_loc_2 <- getSNPID (loc_2, id.type="loc")
15	Species used =    Bos taurus
16	> snp_loc_2
17	[1] Gene_ID    SNP_ID     Location
18	<0 rows> (or 0-length row.names)
19	> geneid_2 <- getGeneID (loc_2, id.type="loc")
20	Species used =    Bos taurus
21	> geneid_2
22	[1] Gene_ID    SNP_ID     Location
23	<0 rows> (or 0-length row.names)
```

From the codes in the text box, we can see that SNP location “265773” (see lines 1-11) can be annotated by FunctSNP, but “265772” (see lines 13-23) can not be recognized and no results are output. Note that FunctSNP can not use the chromosome name as input. This example illustrates that FunctSNP can only annotate the loci included in dbSNP.

The following are screen shots of the HTML outputs of annotating “Chr29, 265773” and “Chr29, 265772” by Snat. Snat provides options to find the nearest SNP from each of queried loci or SNPs within the region from each of queried loci with a

specified distance.

Figure S1.

>>Locus: Chr29,265773

[SNP information]

Option: nearest, Position_of_found_SNP: Chr29,265773, Distance: 0

SNP_ID: rs41576734, Heterozygosity: 0

GeneID	Function	Position_in_codon	Allele	Protein_residue	AA_position	Codon_position_on_mRNA
519580	intron	0			0	

[Gene information]

Gene_Id	Gene_Symbol	Gene_Name	Location	Type_of_gene
519580	HEPHL1	hephaestin-like 1	-	protein-coding

>>Locus: Chr29,265772

[SNP information]

Option: nearest, Position_of_found_SNP: Chr29,265773, Distance: 1

SNP_ID: rs41576734, Heterozygosity: 0

GeneID	Function	Position_in_codon	Allele	Protein_residue	AA_position	Codon_position_on_mRNA
519580	intron	0			0	

[Gene information]

Gene_Id	Gene_Symbol	Gene_Name	Location	Type_of_gene
519580	HEPHL1	hephaestin-like 1	-	protein-coding

A2. Example 2: about the process of annotation

FunctSNP outputs annotation information step by step while Snat produces all results in one step. The following text box is one example of annotating the SNP *rs41576734* using FunctSNP.

```
1   > snpid <- c(41576734)
2   > snp <- getSNPs (snpid)
3   Species used =  Bos taurus
4   > snp
5       SNP_ID Chr Location is_Coding is_Exon           Function Score
6   1 41576734  29   265773         0       0 intron. ex. rs249.     3
7   > geneid <- getGeneID (snpid)
8   Species used =  Bos taurus
9   > geneid
10    Gene_ID   SNP_ID Location
11  1  519580 41576734   265773
12  > geneid <- geneid [, c("Gene_ID")]
13  > pathways <- getKEGG (geneid, "gene")
14  Species used =  Bos taurus
15  > pathways
16  [1] Gene_ID Pathway
17  <0 rows> (or 0-length row.names)
18  > protein <- getProteins (geneid, "gene")
19  Species used =  Bos taurus
20  > protein
21  [1] Gene_ID     Protein_ID   UniProt_ID   Protein_Name
22  <0 rows> (or 0-length row.names)
23  > traits <-getTraits (snpid, "snp")
24  Species used =  Bos taurus
25  > traits
26  [1] SNP_ID     Gene_ID    Trait      QTL_Start QTL_Stop
27  <0 rows> (or 0-length row.names)
```

In the above text box, lines 2-6, lines 7-11, lines 12-17, lines 18-22 and lines 23-27 are operation commands and results for querying for SNP information, gene information, KEGG Pathway information, protein information and QTL information respectively.

For Snat, as one selects the annotation options and submits one query task, all annotation results are generated simultaneously (Figure S2).

Figure S2. HTML outputs of annotation for *rs41576734* using Snat

>>SNP Identifier: rs41576734

[SNP information]

Position: Chr29,265773, Heterozygosity: 0

GeneID	Function	Position_in_codon	Allele	Protein_residue	AA_position	Codon_position_on_mRNA
519580	intron	0			0	

[Gene information]

Gene_Id	Gene_Symbol	Gene_Name	Location	Type_of_gene
519580	HEPHL1	hephaestin-like 1	-	protein-coding

[GeneRIFs]

PubMed_Id	Description
No GeneRIFs Found.	

[Uniprot Protein information]

Accession	Recommended name	Length	Status	Function	Subunit structure	Subcellular location	Post-translational modification
No Information Found.							

[QTL information]

QTL Id	Trait	Type	P-value	F-value	Variance	PubMed_Id	QTL Region
4488	Body weight (birth)	Significant	0.037	11.1	-	17596127	Chr29:0-10024112
5371	Gestation length	Significant	<0.05	2.74	-	19016677	Chr29:0-1907297
5110	Residual feed intake	Significant	0.0013	-	-	18318789	Chr29:0-13920918
5111	Residual feed intake	Significant	0.0011	-	-	18318789	Chr29:0-13920918

[GO information]

Accession	Term	Ontology	Definition
No Information Found.			

[KEGG Pathway information]

Entry	Name	Description	Class
No Information Found.			
