# Supplementary material for: Snat: a SNP annotation tool for bovine by integrating various sources of genomic information
Source: BMC Genet. 2011 Oct 7;12:85. doi: 10.1186/1471-2156-12-85 (PMC3224132; doi:10.1186/1471-2156-12-85)
Supplement: Additional file 3 — Detailed descriptions on annotation results by Snat. An example is provided to give a detailed explanation on the annotation reports. [file 1471-2156-12-85-S3.PDF]

Detailed descriptions on annotation results by Snat

We annotated the SNP *rs109234250*, and the results were showed below in the text box.

>>SNP Identifier: rs109234250
[SNP information]
Position: Chr14,445085 Heterozygosity: 0
GeneID Function Position_in_codon Allele Protein_residue Amino_acid_position Codon_position_on_mRNA
282609 missense 1 A T 231 706
282609 cds-reference 1 G A 231 706
[Gene information]
Gene_Id Gene_Symbol Gene_Name Location Type_of_gene
282609 DGAT1 diacylglycerol O-acyltransferase 1 - protein-coding
[GeneRIFs]
PubMed_Id Description
14983021 K allele, causing an increase in milk fat percentage in the live animal, is characterized by a higher Vmax in producing triglycerides than the A allele.
......
[Uniprot Protein information]
Acc Rec_name Prot_length Status Function Subunit Subcellular PTM
Q8MK44 Diacylglycerol O-acyltransferase 1 489 Reviewed (UniProtKB/Swiss-Prot) Catalyzes the terminal and only committed step in CC triacylglycerol synthesis by using diacylglycerol and fatty acyl CC CoA as substrates. In contrast to DGAT2 it is not essential for CC survival. May be involved in VLDL (very low density lipoprotein) CC assembly (By similarity). NA Endoplasmic reticulum membrane; Multi-pass CC membrane protein. NA
[QTL information]
QTL_Id QTL_Trait QTL_TypeQTL_Pvalue QTL_Fvalue QTL_Variance QTL_PubMed_Id QTL_Region
6214 Age at puberty (EBV) Significant <0.05 - - 18650300 Chr14:0-5235875
......
[GO information]
AccessionTerm Ontology Definition
GO:0004144 diacylglycerol O-acyltransferase activitymolecular_functionCatalysis of the reaction: acyl-CoA + 1,2-diacylglycerol = CoA + triacylglycerol.
......
[KEGG Pathway information]
Entry Name Description Class
bta00561 Glycerolipid metabolism - Bos taurus (cow) NA Metabolism; Lipid Metabolism
......
[HCE information]
No information. The SNP is not in a HCE.
[OMIA information]
No Information Found.

As seen from the above text box, for an individual SNP query, double right angle brackets are placed in the

beginning of the first line of the output, followed by the SNP coordinate or rs-identifier initially input by users. The following contents contain the detailed annotation of the SNP corresponding to the options set for the query. Different assortments of annotations are separated by a pair of square brackets with respective key words enclosed, *e.g.* “[SNP information]”, “[Gene information]”, “[GeneRIFs]”, “[Uniprot Protein information]”, “[QTL information]”, “[GO information]”, “[KEGG Pathway information]”, “[HCE information]” and “[OMIA information]”. For each subsection of annotation results, the headers are printed as the first line, separated by tab characters. The following line(s) is annotation information if any related data is available, otherwise a sentence with first two words “No information” will be printed.

For example, in “[SNP information]” subsection, the first line “Position: Chr14,445085 Heterozygosity: 0” includes two items separated by one tab character, *i.e.* position of the annotated SNP *rs109234250* and its average heterozygosity. It means no information is available if the heterozygosity is 0. The following line “GeneID Function Position_in_codon Allele Protein_residue Amino_acid_position Codon_position_on_mRNA” is the header including 7 items, followed by annotation data. If the queried SNP is not in a gene, the sentence “No information. The SNP is not in a gene” will be shown.

When an item is too long, the sentence may seem messy, *e.g.* “Description” of “[GeneRIFs]” and “Function” of “[Uniprot Protein information]”. However, the sentence contains no tab character, and the whole line can be separated by tab characters into several fields for further processing, which can be achieved by the Perl program raw2TSV.pl provided by Snat.
